# Cassava Starch/Carboxymethyl Cellulose Edible Coating Added of Tocopherol: A Strategy to Preserve the Oxidative Stability of Brazil Nuts

**DOI:** 10.3390/foods13172732

**Published:** 2024-08-28

**Authors:** Danusa Silva da Costa, Kalebe Ferreira Furtado, Ariane Mendonça Kluczkovski, Katiuchia Pereira Takeuchi, Alessandra Santos Lopes

**Affiliations:** 1 Biotechnological Process Laboratory (LABIOTEC), Faculty of Food Engineering (FEA), Institute of Technology (ITEC), Federal University of Pará (UFPA), Rua Augusto Corrêa S/N, Guamá, Belém 66075-900, PA, Brazil; alessalopes@ufpa.br; 2 School of Biotechnology, Institute of Biological Sciences (ICB), Federal University of Pará (UFPA), Rua Augusto Corrêa S/N, Guamá, Belém 66075-900, PA, Brazil; kalebefn7788@gmail.com; 3 Faculty of Pharmaceutical Sciences (UFF), Federal University of Amazonas, Avenida Rodrigo Otavio, n° 6200, Bairro Coroado, Manaus 69067-005, AM, Brazil; 4 Department of Food and Nutrition, Faculty of Nutrition, Federal University of Mato Grosso (UFMT), Cuiabá 78060-900, MT, Brazil; katiuchia.takeuchi@ufmt.br

**Keywords:** antioxidant coating, amazonian fruit, tocopherol, lipid oxidation protection

## Abstract

The aim was to apply a cassava starch/carboxymethyl cellulose blend-based edible coating added to a tocopherol mix to Brazil nuts and evaluate oxidative levels during storage. The edible coatings were prepared from a cassava starch/carboxymethyl cellulose blend and identified as control B (no soy lecithin and no tocopherol mix), L (with soy lecithin and no tocopherol mix), and LT and LT2 (with soy lecithin and tocopherol mix). In the forming solutions of the coatings, stability, viscosity, pH, and color were analyzed. The Brazil nuts were immersed in the solutions for 30 s, dried at 45 °C, and placed in an incubator at 25 °C. At 1, 7, 15, 30, 45, 60, 90, and 120 days of storage, mass loss, the browning index, conjugated dienes and trienes, the oxidative state by official methods, and the accelerated oxidation index were evaluated. The blend-forming solutions B, L, LT, and LT2 showed non-Newtonian and pseudoplastic behavior, excellent resistance to flow, and stability. The diene, triene, iodine value, peroxide value, p-anisidine value, and total oxidation indices showed that the application of the cassava starch/carboxymethyl cellulose blend-based edible coating added tocopherol mix, LT, and LT2 preserved the Brazil nuts up to 90 days of storage at 25 °C. PCA shows that all coatings applied to Brazil nuts promoted oil preservation in some evaluation periods, especially those added with a tocopherol mix. It is concluded that cassava starch/CMC added tocopherol mix edible coatings have a potential application as active packaging for foods, especially nuts.

## 1. Introduction

A coating is one or more thin layers placed on a product, giving it the physical and aesthetic characteristics of the coating material. The conventional process for producing an active coating is based on dissolving or dispersing an active compound in a solvent or matrix that is then applied to the surface of a substrate and dried by evaporation or crosslinking [1].

Active packaging acts directly on the food or in the headspace of the packaging and can act as an absorber of oxygen, moisture, or even the ethylene that results from the ripening process of the food product. Alternatively, the active ingredient can be released/emitted over time in a controlled manner from the package into the headspace or into the food [2,3]. Food packaging has evolved beyond its traditional role of merely containing and protecting food. Now, it actively contributes to enhancing the quality and shelf life of the product by incorporating bioactive compounds, creating what is known as active packaging. This innovative approach allows for the integration of new materials that foster beneficial interactions between the packaging and the food it contains [4].

Coatings, nanofibers, and nanoparticles can play a crucial role in developing active packaging by providing enhanced protection to food products against oxidation caused by external factors. These advanced materials can be engineered to create barriers that inhibit the permeation of oxygen, moisture, and other environmental elements, effectively prolonging the freshness and quality of the food. Additionally, the incorporation of bioactive molecules such as phenolic compounds, tocopherols, carotenoids, and others into these materials can further boost their antioxidant properties, offering a robust defense against spoilage and significantly extending the shelf life of various food products [5].

Antioxidant agents are used in food packaging to inhibit or slow down oxidation reactions that affect food quality. They react with reactive oxidant species (e.g., peroxides, superoxides, and hydroxyl radicals) to slow down or block oxidation reactions in food products. The active agents are released from the packaging material into the headspace by vaporization, migration, or diffusion into the food [6,7]. Tocopherols are used as powerful antioxidants [8] and applied in polymers in various research areas, for example, in cosmetics [9], medicine [10], as well as in the food sector [11,12,13]. Tocopherol, generally known as vitamin E, is an excellent radical-chain breaker in unsaturated fatty foods and is a lipid-soluble antioxidant that may be derived from food sources such as palm oil, sunflower oil, and soybeans. Furthermore, tocopherols are employed as antioxidants in food products in four different forms (α, β, γ, and δ), and their effectiveness decreases as follows: δ > γ > β > α [14,15].

The Brazil nut is one of the most important non-timber forest products, and the livelihood of thousands of traditional families depends on its commercialization [16,17]. Nuts and seeds are sources of fatty acids, phytosterols, fiber, and polyphenols and are low in sodium [18]. The Brazil nut is composed of proteins (16.03 g 100 g^−1^), lipids (58.52 g 100 g^−1^), carbohydrates (19.61 g 100 g^−1^), and ash (3.35 g 100 g^−1^) [19], besides 5.24 µg g^−1^ of selenium [20]. According to the Instituto Brasileiro de Geografia e Estatística—IBGE, the production of Brazil nuts in 2021/2022 was 38,160 tons, predominantly in the North region, whose production was 35,964 tons, and the state of Para was responsible for the production of 8807 tons in this period [21].

Due to the high concentration of unsaturated fatty acids in the Brazil nut, it becomes quite perishable, especially when exposed to trading for an extended period, because it is subjected to high temperature and relative humidity conditions and is exposed to oxidative processes that can contribute to the loss of nutrients and the occurrence of rancid odor and flavor in Brazil nuts [22,23].

Some studies have evaluated the effect of storage conditions on oxidative changes in Brazil nuts. These include, for example, the studies by Ribeiro et al. [21], who packed Brazil nuts in kraft paper bags and stored them at temperatures of −15, 2 and ambient ranging from 18 to 25 °C, and Casagrande et al. [24], who stored Brazil nuts in transparent and opaque glass and plastic containers at temperatures of 11 and 24 °C.

In the study by Leme et al. [25], thermoplastic starch/poly (butylene adipate-co-terephthalate) (TPS/PBAT) packaging containing curcumin and water-soluble pine nuts and Brazil nuts were packaged and packed in airtight jars stored at 10, 25, and 50 °C. Lipid oxidation was determined at 0, 5, 10, 15, and 30 days of storage. However, no studies have been found on monitoring the lipid oxidation profile of Brazil nuts packed with an edible coating based on cassava starch/carboxymethylcellulose incorporated with tocopherols and stored for different lengths of time.

Therefore, developing an active edible coating that contains antioxidants may be a protective strategy for Brazil nuts and minimize lipid oxidation levels. This work aimed to apply a cassava starch/carboxymethyl cellulose blend-based edible coating with a tocopherol mix to Brazil nuts and evaluate oxidative levels during storage.

## 2. Materials and Methods

The Brazil nuts (kindly provided by the Mutran export company, Belém, Pará, Brazil) were registered at Sisgen—National System for Managing Genetic Heritage and Associated Traditional Knowledge, nº A9659A8/2023. The Brazil nuts, according to information provided by the exporting Mutran Company, were from the 2022 harvest, collected from January to June, and had been dried at 50 °C for 8 h and dehydrated at 70 °C for 18 to 20 h until the donation, which occurred in December 2022. The Brazil nuts went through biometry and weighing for classification according to the criteria of Brasil [26]. They were classified as peeled or processed nuts, small, measuring on average 27.10 cm in height, 12.85 cm wide, and 10.96 cm thick, and weighing 2.07 g.

The material used to formulate the solutions for the formation of the coatings, all of which were food-grade: carboxymethyl cellulose (lot 25249 Arcolor, São Paulo, Brazil), cassava starch (lot HW294, Amafil, Cianorte, PR, Brazil), sorbitol in solid form (powder), (lot 250FU46, Ingredientes on-line, São Paulo, Brazil), soy lecithin (lot 2021105, Ingredientes on-line, São Paulo, Brazil), and tocopherol mix (mix of tocopherols—α-tocopherol: 10–20%; β-tocopherol: 1–3%; γ-tocopherol: 45–65%; δ-tocopherol: 12–26%) (Vonplex E^®^ L., São Paulo, Brazil).

### 2.1. Measures and Method of Preparation of Cassava Starch/Carboxymethyl Cellulose Edible Coatings

**Figure 1:**
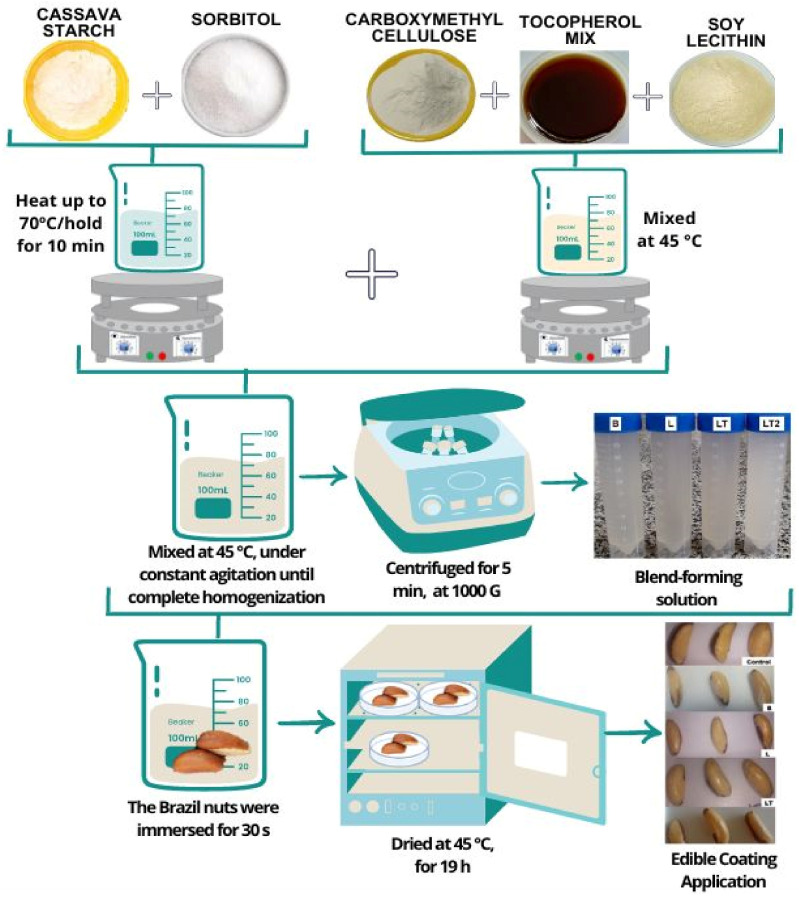
Preparation of edible coatings.

The edible coatings were prepared using the methodology of Tongdeesoontorn et al. [27] and Wu et al. [28]. Laboratory pre-tests were used to define the formulations. Four formulations called B, L, LT, and LT2 were prepared. Fixed amounts of cassava starch + carboxymethyl cellulose + sorbitol and water were used (3 g + 1.5 g + 0.2 g + 100 g, respectively), and the soy lecithin was added in the proportion of 20% to the tocopherol mix in the formulation L, LT, and LT2. The formulation L was prepared with the same amount of lecithin as LT2. The amount of tocopherol mix was added to the blends in the following proportions: B—0, L—0, LT—0.0005 g, and LT2—0.010 g. For the preparation of edible coatings (Figure 1), the solution of cassava starch, sorbitol, and 70% of the total volume of water was stirred (Quimis Q261-22, Diadema, SP, Brazil), raising the temperature to 70 °C, and maintained for 10 min to induce the gelatinization of the starch to form a paste. In another container, the carboxymethyl cellulose, the tocopherol mix, soy lecithin, and 30% of the total volume of water were mixed at 45 °C, and the starch paste was added to this mixture under constant agitation until complete homogenization to form a blend. The blends were subjected to centrifugation in a centrifuge (Quimis Q222, Diadema, SP, Brazil) for 5 min at 1000 G to remove air bubbles and stored until the moment of application, reserving part of the solution for the viscosity, centrifugation, creaming, pH, and antioxidant activity tests.

### 2.2. Tests of the Blend-Forming Solutions Formatting Edible Coatings

#### 2.2.1. Rheology

The rheological parameters of the solutions B, L, LT, and LT2 were obtained at a temperature of 45 °C using a rheometer (Physica, MCR 101, Ostfildern, Germany), with a deformation rate of 1 to 500 s^−1^ in cone-plate geometry. The data were fitted to Newton, power law, and Herschel–Bulkley rheological models using Equations (1), (2), and (3), respectively. The coefficient of determination (R^2^), the reduced chi-squared value (χ^2^), and the root mean square error (RMSE) were the parameters used to evaluate the fits.

(1) $$\sigma =\dot{\gamma }.n$$

(2) $$\sigma =k.{\dot{\gamma }}^{n}$$

(3) $$\sigma =\sigma_{0}+k.{\dot{\gamma }}^{n}$$

where σ—shear stress (Pa); γ—shear rate (s); k—consistency index (Pa·s^n^); n—fluid behavior index (dimensionless); σ_0_—residual stress (Pa).

#### 2.2.2. Centrifugation

In a centrifuge (Spinlab Scientific SL 5-GR, Shanghai, China), test tubes containing 2 g of each solution B, L, LT, and LT2 were subjected to 30 min cycles in rotation of 178, 1113, and 2183 G [29]. The volumes of the supernatant were quantified at each cycle.

#### 2.2.3. Creaming Index

Immediately after preparation, 50 mL of each of the B, L, LT, and LT2 solutions was transferred to closed 50 mL graduated beakers and kept at a controlled temperature of 25 °C. If no sample showed phase separation in the first few hours, the volume of the aqueous phase was quantified every 24 h until the seventh day of testing [30]. The stability was measured through the height of the upper phase, the creaming index described by Equation (4).

CI (%) = (H/H_0_) ∗ 100 (4)

where H_0_—initial height of the lower phase and H—initial height of the upper phase after 24 h.

#### 2.2.4. pH

The pH of solutions B, L, LT, and LT2 was measured using a bench pH meter (Luca 210P, Campinas, SP, Brazil) using method n°. 981.12 [31].

#### 2.2.5. Color Parameters

The color measurements of the edible coating solutions B, L, LT, and LT2 were carried out using a colorimeter (Konica Minolta Sensing IC., CR-400, Sakai, Japan). The parameters L* lightness, a* chromaticity, b* chromaticity, C*-chroma, and Hue angle (h°) were evaluated.

#### 2.2.6. Antioxidant Activity

To analyze the antioxidant activity using the DPPH• (1,1-diphenyl-2-picrylhydrazyl) method of the edible coating solutions B, L, LT, and LT2, the methodology described by Mensor et al. was followed [32]. Using a UV/vis spectrophotometer (Bel Engineering UV-M51, Monza, Italy), absorbance was measured at 520 nm, and total antioxidant activity was calculated using Equation (5). Ethanol was used as a blank, and the extraction solution was used as a control.

(5) $$AA\left(\%\right)=100-[(ABSsample-ABSwhite)/ABScontrol]*100$$

### 2.3. Determination of the Oxidation Level of Brazil Nuts

To evaluate the oxidation level of the Brazil nuts, the nuts were immersed for 30 s in the blend-forming solutions B, L, LT, and LT2, deposited in non-stick trays, and dried at 45 °C in the oven (Lucadema Científica, 80/336, São José do Rio Preto/SP, Brazil) for 19 h. After drying, the nuts, coated and uncoated, were conditioned in a B.O.D. (347 CD, Fanem, Brazil).

To monitor the lipidic oxidation profile of the Brazil nuts, coated or not, in the periods 1, 7, 15, 30, 45, 60, 90, and 120 days of conditioning at 25 ± 0.5 °C, the mass loss, the browning index, conjugated dienes and trienes, the oxidative state by official methods, and the accelerated oxidation index were determined. For the analyses, the oil was extracted from the Brazil nuts by cold pressing using an ERT 60III press (N.S. 597, Vinhedo, SP, Brazil).

#### 2.3.1. Loss of Fresh Mass

The mass loss of the Brazil nuts was carried out by gravimetry, the mass loss of the Brazil nuts was expressed in % [33].

#### 2.3.2. Index of Browning

The evaluation of the index of browning (IE) of the Brazil nuts uncoated and coated was according to Palou et al. [34] and Equations (6) and (7).

IE = [100 (x − 0.31)/0.172](6)

x = [(a + 1.75*L)/(5.645*L + a − 3.012*b)](7)

where L = luminosity and the chromaticities a (-a: green and +a: red) and b (-b: blue a +b: yellow).

#### 2.3.3. Determination of Conjugated Dienes and Trienes

The determination was by UV light absorptivity at wavelengths 232 and 270 nm (conjugated dienes and trienes, respectively) according to method Ch 5-91 [35]. The calculations were performed using Equation (8).

(8) $$E_{1cm}^{1\%}=(A/C)$$

where A—absorbance at λ of 232 nm for the conjugated dienes and λ of 270 nm for the conjugated trienes; C—concentration of the solution (g. 100 mL^−1^).

#### 2.3.4. Quantification of Oxidative Status by Official Methods

The lipid oxidation degree of the Brazil nuts was evaluated using the free fatty acid (FFA—AOCS Ca 5a-40) and iodine value (IV—AOCS Cd 1-25) [35]. The formation of primary and secondary products (carbonyl products) from lipid oxidation was evaluated. The primary products (LOOH) were monitored using the peroxide value (PV—AOCS Cd 8b-90) [35]. The secondary products were determined using the p-anisidine value (p-AnV—Cd 18-90) [35], which was determined spectrophotometrically at 350 nm. Based on p-AnV and PV, the overall oxidation rates of coated and uncoated Brazil nuts during storage were calculated as total oxidation (TOTOX = 2PV + p-AnV) [36].

#### 2.3.5. Accelerated Oxidation—Induction Time

The accelerated oxidation test of Brazil nuts was conducted using the AOCS Cd 12b-92 method [37] and Rancimat 743 equipment (Metrohm Schweiz AG, Zofigen, Switzerland). The test was carried out using 3.0 ± 0.1 g of the oil sample with an airflow of 15 L h^−1^ at 110 °C. The induction time (IT) was expressed in hours.

##### Oxidation Kinetics of Brazil Nuts

The zero-, first-, and second-order kinetic constants for the oxidation of Brazil nut oil were calculated using the kinetic models shown in Table 1.

For the adjustment of the mathematical models, non-linear regression analysis was performed using the Solver computer software for Microsoft Excel 2016, using the Gauss–Newton method. The model’s performance was evaluated using statistical parameters such as coefficient of determination (R^2^), chi-square (χ^2^), and root mean square error (RMSE).

The half-life time (θ (1/2)) was calculated using Equation (12) for the zero-order kinetic model, Equation (13) for the first-order kinetic model, and Equation (14) for the second-order kinetic model. The value of k used was that of the kinetic model that best fits the kinetic experimental data.

(12) $$\theta_{(1/2)}=A_{0}/2_{k}$$

(13) $$\theta_{(1/2)}=ln2/k$$

(14) $$\theta_{(1/2)}=1/kA_{0}$$

### 2.4. Statistical Treatment of Data

All the tests were carried out in triplicate. For a better understanding, the statistical treatment of the data will be listed:

a. The rheological data of the solutions B (no soy lecithin and no tocopherol mix), L (with soy lecithin and no tocopherol mix), and LT and LT2 (with soy lecithin and tocopherol mix) were adjusted to Newton, power law, and Herschel–Bulkley models to evaluate the behavior of the emulsions and graphs was be generated using Oring Pro 9.6.5.169 software. The Levene test checked the homoscedasticity of the fit parameters, and the normality of the data was evaluated using the Shapiro–Wilk test. For the data that were considered normal, the Tukey test was performed (*p* ≤ 0.05) for variance analysis and comparison of means of the parameters using the statistical program Jamovi 2.3.26 [38].
b. The data from the centrifugation data, pH and color parameters of the solutions, and the results of the tests to evaluate the lipid oxidation of the Brazil nuts were checked for homoscedasticity using the Levene test, the normality of the data was evaluated using the Shapiro–Wilk test; the data were considered normal; the Tukey test (*p* ≤ 0.05) was performed for analysis of variance and comparison of the means of the parameters using the Jamovi 2.3.26 statistical program [38].
c. The Cremeation results were submitted to linear regression using Microsoft’s Excel 2010^®^ spreadsheet. The Rancimat results were adjusted to the mathematical models for evaluating oxidation kinetics, also using Excel 2010^®^.
d. The physicochemical parameters and the induction time obtained in the Rancimat test to assess the level of oxidation of Brazil nuts with and without coating were subjected to a principal components analysis (PCA), and a biplot was generated to explain the variability of the data. To analyze the similarity of the results obtained, a hierarchical cluster analysis (HCA) was carried out, and a cluster dendogram graph was generated using the software Past4.03 [39].

## 3. Results and Discussion

### 3.1. Rheological Behavior of Blend-Forming Solutions

**Figure 2:**
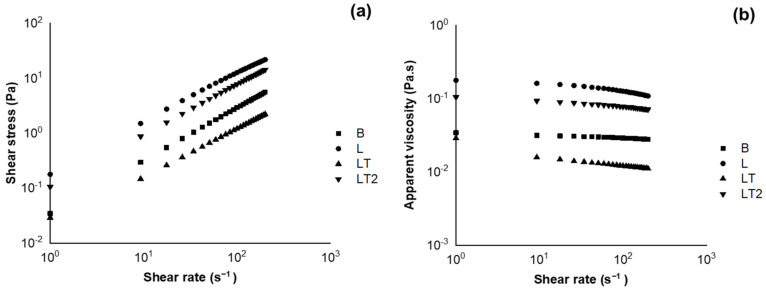
Flow curves of blend-forming solutions B (no soy lecithin and no tocopherol mix), L (with soy lecithin and no tocopherol mix), and LT and LT2 (with soy lecithin and tocopherol mix) at 45 °C—(**a**) shear stress vs. shear rate and (**b**) apparent viscosity vs. shear rate.

Flow curves were drawn to assess the rheological behavior of the blend-forming solutions. The data generated by the test make it possible to test rheological models that make it possible to visualize information such as the consistency index, for example, which indicates the degree of flow, i.e., how much the fluid flows, which can reflect industrially how much the tested fluid resists flow if subjected to a pipe. In addition, assessing the viscosity of fluids allows them to be classified as Newtonian (constant viscosity) or non-Newtonian (viscosity varies with the magnitude of the shear rate). Figure 2 shows the flow curves of blend-forming solutions B (no soy lecithin and no tocopherol mix), L (with soy lecithin and no tocopherol mix), and LT and LT2 (with soy lecithin and tocopherol mix) at 45 °C—(a) shear stress vs. shear rate and (b) apparent viscosity vs. shear rate.

Figure 2a shows that the blend-forming solutions B, L, LT, and LT2 continuously increased shear stress according to the increase in the strain rate in all solutions; this behavior is characteristic of non-Newtonian fluids. The increase in the strain rate may have caused a collapse in the solutions’ structure, leading to a more aligned arrangement of the molecules [40].

In Figure 2b, it is observed that in the blend-forming solutions B, L, LT, and LT2, the results showed apparent viscosity decreases with an increase in shear rate. This may have occurred because the shear rate disrupted the fluids’ intermolecular junctions, preventing reformation and decreasing the viscosity [41,42].

The blends L and LT2, with a higher concentration of lecithin in their formulation, showed higher viscosity; it is inferred that soy lecithin caused greater resistance to the flow of these fluids, but the concentration of the emulsifier did not favor the flow in the blend. LT2, in addition to the soy lecithin content, also had the highest concentration of tocopherol mix, forming intermolecular hydrogen bonds and causing reinforcement in the intermolecular network, making the blend more viscous [42,43]. Fluid flow behavior can affect various film-forming solution properties, such as thickness, design sizing, application form, spreadability, and mechanical properties [42,44].

The behavior of the flow curves can be attributed to the excellent interaction between the compounds in the solution forming the coatings, such as cassava starch + carboxymethyl cellulose + sorbitol + soy lecithin + tocopherol mix + water. In addition, the LT blend-forming solution had the lowest values throughout the test, so at the minimum concentration of the tocopherol mix, the blend-forming solution had the lowest resistance to flow.

Table 2 shows the results of the fit parameters of the Newton, power law, and Herschel–Bulkley models to the rheological behavior of the blend-forming solutions B (no soy lecithin and no tocopherol mix), L (with soy lecithin and no tocopherol mix), and LT and LT2 (with soy lecithin and tocopherol mix).

The power law and Herschel–Bulkley models were the ones that best fit the rheological behavior of all blend-forming solutions. When using the power law and Herschel–Bulkley models to describe the structural behavior of samples, a high correlation coefficient (R^2^ > 0.9999) was obtained (Table 2). The *n* values of all samples were lower than 1, meaning that they were pseudoplastic fluids. However, all blend-forming solutions showed an *n* value close to 1, indicating closer to Newtonian fluids and a lower influence of shear rate on viscosity. Thus, the solution was more stable [42,45]. It is worth mentioning that a pseudoplastic fluid is a fluid in which the apparent viscosity decreases according to the shear rate and has *n* < 1. This is precisely what happened with the blend-forming solutions produced in this study, which becomes clear when looking at the behavior of the curves in Figure 2b and the values of *n* in Table 2. It is worth mentioning that a Newtonian fluid with *n* above 1 presents itself graphically in a linear fashion. Thus, the fluids in this study had values below 1, as already mentioned, and Figure 2b shows that the flow curves are slightly inclined, making it possible to characterize the fluids under study as non-Newtonian fluids.

Another essential factor to mention was the behavior index, which explains the results of the flow curves since the LT blend-forming solution had a lower K value than the other coating-forming blend solutions. It can be inferred that the concentration of the tocopherol mix added to this blend made it less resistant to flow due to the good interaction between the formation compounds in the blend-forming solution, especially at the lowest concentration of the tocopherol mix.

**Figure 3:**
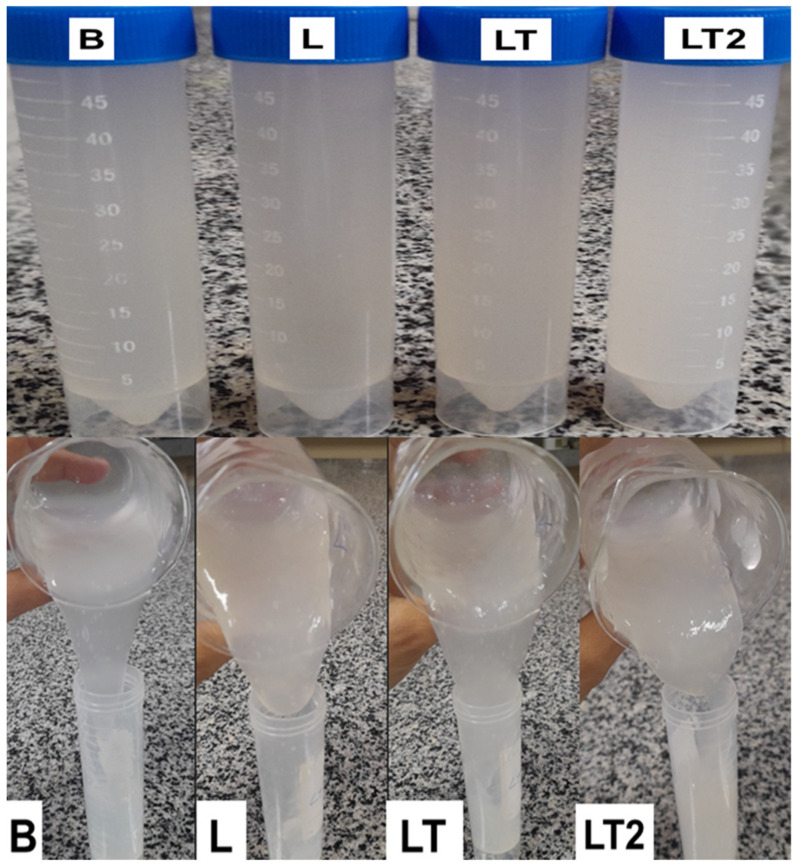
Blend-forming solutions B, L, LT, and LT2.

Figure 3 shows the blend-forming solutions B, L, LT, and LT2.

The blend-forming solutions B, L, LT, and LT2 are shown in Figure 3. Visually, it can be seen that the mixtures forming the coatings that contained soya lecithin in their formulation had a thicker appearance than formulation B, so one would imagine that they might be more resistant to flow, but only solutions L and LT2 were more resistant to flow. While LT was surprisingly easier to flow than formulation B, it can be inferred that the lower concentration of emulsifier and tocopherol mix promoted a good interaction in the composition of this formulation, contributing to the greater fluidity of this formulation, corroborating the k value shown in Table 2 for the power law model, which was lower than the result for the other formulations.

### 3.2. Centrifugation and Creaming Index

The centrifugation test stresses the sample to simulate increased gravitational force, anticipating signs of sample instability, such as phase separation [46]. The creaming index is important for checking the destabilization of emulsions, as phase separation can occur due to the difference in densities [47].

No phase separation occurred in the centrifugation test or the creaming test, and data were not shown. This behavior can be attributed to the good interaction of the ingredients, which promoted the stability of all the solutions. The rheological behavior shown in the power law model (Table 2) corroborates this since it showed behavior index values close to 1, demonstrating the high stability of all the blend-forming solutions.

### 3.3. pH, Color Parameters and Antioxidant Activity

Table 3 shows the pH, color parameters, and antioxidant activity of the blend-forming solutions B (no soy lecithin and no tocopherol mix), L (with soy lecithin and no tocopherol mix), and LT and LT2 (with soy lecithin and tocopherol mix).

Table 3 shows that the blend-forming solutions L and LT were similar according to Tukey’s test and significantly different according to Tukey’s test (*p* ≤ 0.05) compared to B and LT2. The pH results showed that L, LT, and LT2 were superior to formulation B, demonstrating the influence of soy lecithin in these formulations. Meanwhile, the LT2 solution had the highest pH value, i.e., the higher concentration of the tocopherol mix caused an increase in the pH value. It can be said that this result did not adversely affect the polymer matrix or the stability of the solutions because, as already mentioned, all the solutions showed high stability in the creaming and centrifugation tests, demonstrating the good interaction of the cassava starch/carboxymethyl cellulose combination with sorbitol and water. Bai et al. [48] reported that polysaccharides are more stable than proteins due to variations in pH, ionic strength, and temperature increase. So, the blend-forming solutions did not suffer so much change due to the pH results, as they all presented high stability without phase separation, as mentioned above.

Another important factor regarding the pH of an edible coating-forming solution is that the human body has extreme physiological pH variations throughout the gastrointestinal tract, ranging from pH 2 in the stomach to pH 7 in the colon and up to pH 8.2 in the lower duodenum [49]. So, the pH values found, which ranged from 6.05 to 6.53 when entering the gastrointestinal tract, could be digested without gastric damage by humans.

The color parameters (Table 3) showed that the luminosity parameter of all samples tended slightly toward white. The chroma values were low (achromatic), revealing weak or diluted coloration; it is clear when observing the chromaticity parameters a* and b*. The hue angle reveals that all the solutions had a slightly greenish-yellow tonality. Adding a tocopherol mix promoted a greater tonality in the blend-forming solutions LT and LT2.

The antioxidant activity (Table 3) revealed that the blend-forming solutions LT and LT2 (with soy lecithin and tocopherol mix) showed good antioxidant activity, as they preserved values of 58.40 and 81.69, respectively, after the heating process. This means that a really active, bioactive polymer can be applied to the surface of Brazil nuts.

### 3.4. Evaluation of the Oxidation Level of Brazil Nuts

#### 3.4.1. Loss of Fresh Mass of the Brazil Nuts Uncoated and Coated during Storage Time

**Figure 4:**
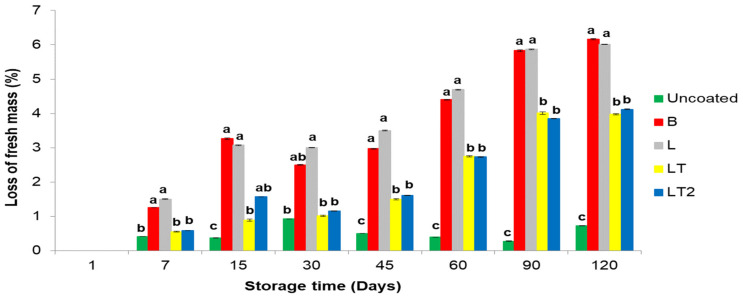
Loss of fresh mass of Brazil nuts uncoated and coated with a coating of the cassava starch/CMC control B (no soy lecithin and no tocopherol mix), L (with soy lecithin and no tocopherol mix), and LT and LT2 (with soy lecithin and tocopherol mix) during storage time. Means followed by the same letter at each storage time did not differ significantly by Tukey’s test at the 5% level.

The loss of fresh mass for nuts is a limiting factor for their preservation and marketing, as the nuts have a dry/shriveled appearance and consequently losses that can be in nutritional aspects, on the oxidative level of the nut. Figure 4 shows the loss of fresh mass of Brazil nuts uncoated and with coating of the cassava starch/CMC control B (no soy lecithin and no tocopherol mix), L (with soy lecithin and no tocopherol mix), and LT and LT2 (with soy lecithin and tocopherol mix) during storage time.

The loss of fresh mass of Brazil nuts uncoated and coated with coating of the cassava starch/CMC control B (no soy lecithin and no tocopherol mix), L (with soy lecithin and no tocopherol mix), and LT and LT2 (with soy lecithin and tocopherol mix) (Figure 4) showed significant difference by Tukey’s test (*p* ≤ 0.05) between uncoated and coated Brazil nuts at almost all storage times, B and L coated nuts were similar while LT and LT2 were similar to each other. And showed that the uncoated Brazil nuts had the lowest weight loss when compared to the coated Brazil nuts, but even if this had happened, it was observed that the mass losses of the Brazil nuts coated with LT and LT2 (with soy lecithin and tocopherol mix) minimized the losses suffered by control B (no soy lecithin and no tocopherol mix) and L (with soy lecithin and no tocopherol mix). The loss of fresh mass presented by coated Brazil nuts is important because, for fresh vegetables, it is estimated that a loss of between 5 and 10% is tolerable so that the vegetable does not become shriveled or wrinkled [50]. Thus, the coatings containing the tocopherol mix preserved the nuts, as they represented only 4% of mass loss after 120 days of storage.

#### 3.4.2. Index of Browning of the Brazil Nuts Uncoated and Coated during Storage Time

**Figure 5:**
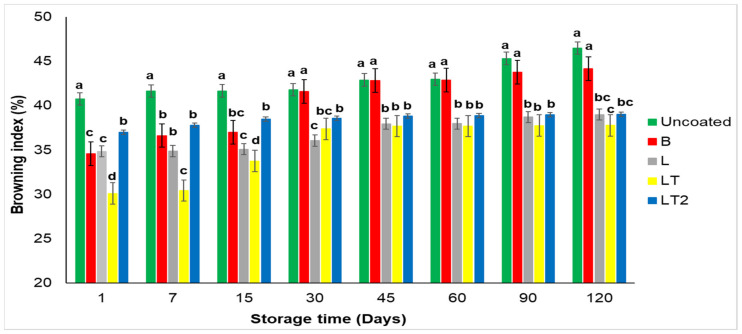
Browning index of Brazil nuts uncoated and coated with a coating of the cassava starch/CMC control B (no soy lecithin and no tocopherol mix), L (with soy lecithin and no tocopherol mix), and LT and LT2 (with soy lecithin and tocopherol mix) during storage time. Means followed by the same letter at each storage time did not differ significantly by Tukey’s test at the 5% level.

Lipid oxidation generates volatile compounds that cause rancidity, while the Maillard reaction can alter the color of dried foods and decrease their nutritional value. These reactions are interrelated [51]. Figure 5 shows the browning index of Brazil nuts uncoated and coated with the cassava starch/CMC control B (no soy lecithin and no tocopherol mix), L (with soy lecithin and no tocopherol mix), and LT and LT2 (with soy lecithin and tocopherol mix) during storage time.

The browning index of Brazil nuts uncoated and coated with coating of the cassava starch/CMC control B (no soy lecithin and no tocopherol mix), L (with soy lecithin and no tocopherol mix), and LT and LT2 (with soy lecithin and tocopherol mix) (Figure 5) showed significant difference by Tukey’s test (*p* ≤ 0.05) between the uncoated Brazil nuts and the coated nuts at storage times 1, 7 and 15 and was similar to the B-coated nuts at the other storage times, while the L, LT and LT2 coated nuts were similar to each other at times 45, 60 and 90, and that all the coated nuts showed a degree of preservation of the browning level on some of the storage days. It can also be seen that the nuts coated with LT showed more stable values after 30 days of storage. Furthermore, it should be noted that the coatings that have a higher soy lecithin content in their formulation were those that minimized the browning of the nuts the most, and the nuts coated with LT2 were the best preserved, as they increased a browning index of just 2% upon arrival within 120 days of storage. This result shows the excellent interaction of cassava starch/carboxymethyl cellulose combined with sorbitol and water. Polyphenol oxidase activity is considered to be the main cause of browning, and antioxidants can inhibit this activity effectively [52,53].

#### 3.4.3. Physical and Chemical Parameters of Oil of the Brazil Nuts Uncoated and Coated during Storage Time

Table 4 shows the results of the quality parameters and induction time of the oil of the Brazil nuts uncoated and coated with the coatings of cassava starch/CMC control B (no soy lecithin and no tocopherol mix), L (with soy lecithin and no tocopherol mix), and LT and LT2 (with soy lecithin and tocopherol mix) during storage time.

##### Dienes (K232) and Trienes (K268) Values

It can be seen that the diene and triene indices (Table 4) of the oil of the Brazil nuts uncoated from day 7 of storage showed a sharper increase than the oil of the Brazil nuts coated, which indicates the presence of primary oxidation products and a high presence of secondary products in the sample. It can be seen that the oil of the Brazil nuts coated was the most preserved throughout the storage period, i.e., these coatings slowed down the oxidation reaction of the coated nuts. Sartori et al. [54] found diene values much higher than those observed in the present study in Brazil nuts packaged in amber and transparent glass, stored for 5 months; all values were higher than 3.

##### Free Fatty Acids (FFA) Value

As for free fatty acids (Table 4), in general, the oil of the uncoated Brazil nuts and those coated with LT2 had the smallest changes in value when compared to the others. However, at 120 days of storage, this FFA increased in the oil of the nuts coated with LT and LT2. This increase can be attributed to the content of the mixture of tocopherols present in the coatings, which may have oxidized during the 120 days of storage.

Even with these results, the maximum value recommended by the National Health Surveillance Agency and the Codex Alimentarium Commission for cold-pressed crude oils is a maximum of 4 mg KOH·g^−^^1^ [55,56], i.e., only the Brazil nut oil coated with LT was higher than this value on the last day of storage. Machado et al. [57] found that in a study with Brazil nut oil from the south of Amazonas without indicating the form of extraction or preservation, 5.56 mg KOH·g^−^^1^ was higher than the values obtained in the present study on all evaluation days. The industry widely uses FFA to monitor the oxidative stability of oil. However, recent studies have shown that free fatty acids may not reflect oxidative stability, as bound fatty acids are the preferred substrate for oxidation [58].

##### Iodine Value (IV)

The iodine value results (Table 4) from the 15 days of storage onwards showed that the oil of uncoated and coated Brazil nuts B maintained higher indices than the oil of the coated Brazil nuts L, LT, and LT2, with the exception of time 90 of storage when the oil of coated Brazil nuts B was lower than L and LT. However, Tukey’s test showed that the oils of the coated Brazil nuts B and LT2 were statistically similar (*p* ≤ 0.05). At 90 and 120 days of storage, the oil of the Brazil nuts coated by LT showed higher values. This index shows the degree of oil saturation, demonstrating that the oil from Brazil nuts coated L, LT, and LT2 was more saturated than the oil from Brazil nuts uncoated and coated B on the storage days mentioned here.

The IV is important in indicating the oxidative stability of edible oils since a high degree of unsaturation in the oil increases its susceptibility to lipid oxidation. On the other hand, a low iodine value in oils is associated with good quality and longer shelf life [59,60]. It can be seen that the values found in this study were low for Brazil nut oil, as Machado et al. [57] found a value of 108.81 g·100 g^−^^1^ for the Brazil nut oil evaluated in their study, so it can be said that Brazil nut oil with and without coating showed good oxidative stability based on the iodine index over the storage period at 25 °C.

##### Peroxide Value (PV)

In the peroxide value (Table 4), the uncoated and coated Brazil nut oil was within the limits recommended by ANVISA and Codex Alimentarium, in which the maximum value for cold-pressed crude oils is no more than 15 meq kg^−^^1^ [55,56], throughout the storage period, except at 120 days when the LT2 coated nut oil was higher than established. The oil of the Brazil nuts was preserved uncoated and coated with B, L, and LT for the entire period evaluated without harming consumer health. Only the oil of the coated Brazil nuts with LT2 showed a value of 17.62 meq kg^−^^1^, which is higher than that recommended by legislation.

Machado et al. [58] found a peroxide value of 17.26 meq kg^−^^1^ for the Brazil nut oil evaluated in their study, so it can be said that Brazil nut oil with and without coating showed good oxidative stability based on PV for 90 days of storage at 25 °C. Sartori et al. [54] found PV values close to 3, 3.5, and 7 meq kg^−^^1^ at 0, 30, and 60 days, respectively, for Brazil nut oil stored in amber and transparent glass, but the authors observed that after 90 days of storage, there was a differentiation since the PV was close to 8 meq kg^−^^1^ for Brazil nut oil stored in amber glass at 90 and 120 days, while for Brazil nut oil stored in transparent glass, the values were close to 10.5 and 15 meq kg^−^^1^ at 90 and 120 days, respectively. PVs are close to those observed in this study for the same storage times.

##### p-Anisidine Value (p-AnV)

The p-anisidine value (Table 4) measures secondary degradation products, measuring the amount of non-volatile aldehydes in the oil [61]. ANVISA and Codex Alimentarium recommend no limits for p-AnV. However, values were observed in the first storage time close to 8, which decreased after packaging in coatings and in an environment with controlled temperature (25 ± 0.5 °C).

##### Total Oxidation (TOTOX)

The total oxidation (Table 4) of the oil of the uncoated and coated Brazil nuts showed values above 10 throughout the storage period. TOTOX is calculated by associating PV (primary oxidation products) with p-AnV (secondary oxidation products). Hydroperoxides (peroxides) are unstable and do not provide a reliable picture of an oil’s oxidative stability. Therefore, a TOTOX lower than 10 indicates better oil quality [62]. Values greater than 10 indicate the oil’s low stability [63].

Oils with a high degree of unsaturation are more susceptible to oxidation [64]. Brazil nuts have a high content of polyunsaturated fatty acids [64]. We evaluated the fatty acid profile of Brazil nuts and found that they contained 75.34 g·100 g^−^^1^ of polyunsaturated fatty acids. Kornsteiner-Krenn et al. [65] evaluated the fatty acid composition of nuts and observed a polyunsaturated fatty acid content of 55.7 g·100 g^−^^1^ in Brazil nuts. Despite not having analyzed the fatty acid composition of the Brazil nuts used in this study, the fatty acids present in their composition contributed greatly to the oxidation state of the nuts. The nuts under study already had some degree of oxidation when the coatings were applied, which may have contributed to the results.

#### 3.4.4. Induction Time and Modeling Oxidation Kinetics of Oil of the Brazil Nuts Uncoated and Coated during Storage Time

**Figure 6:**
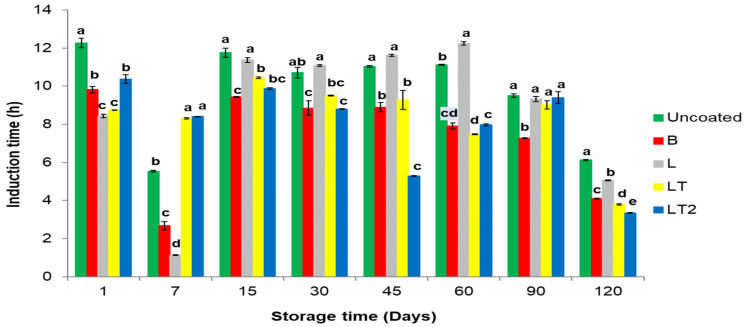
Induction time of oil of Brazil nuts uncoated and coated with a coating of the cassava starch/CMC control B (no soy lecithin and no tocopherol mix), L (with soy lecithin and no tocopherol mix), and LT and LT2 (with soy lecithin and tocopherol mix) during storage time. Means followed by the same letter at each storage time did not differ significantly by Tukey’s test at the 5% level.

Figure 6 shows the induction time of oil of the Brazil nuts uncoated and coated with coating of the cassava starch/CMC control B (no soy lecithin and no tocopherol mix), L (with soy lecithin and no tocopherol mix), and LT and LT2 (with soy lecithin and tocopherol mix) during storage time.

The development of undesirable compounds resulting from lipid oxidation is an important problem that needs to be solved to prolong the shelf life of oils, fats, and fatty foods. To minimize the damage caused to food by oxidation and preserve the oxidative stability of oils, the oilseed industry uses antioxidants [66]; however, in this study, the oxidant was not added directly to the oil but to the coatings that covered the oilseed. The antioxidant was only added to the LT and LT2 coatings.

It can be seen that during the induction time (Figure 6), showed a significant difference by Tukey’s test (*p* ≤ 0.05) between the samples at almost all storage times, only at the 90-day storage time was there a similarity between the oil from the uncoated and L, LT and LT2 coated Brazil nuts. The oil of the coated Brazil nuts underwent some changes in the oxidative state, although the use of the coatings largely preserved them. However, at 120 days, the Brazil nuts coated with B, LT, and LT2 had the shortest induction times, corroborating the PV results at the same storage time.

The Rancimat test can be used to evaluate the oxidative stability phases of vegetable oils [67,68]. The Rancimat test by conductimetry determines the oil’s resistance time to oxidation [69,70]. At 7 days of storage, the uncoated and B- and L-coated Brazil nut oils showed a sharp drop in IT values due to the absence of the antioxidants protecting the uncoated and B- and L-coated packaged Brazil nut oils. At 45 days of storage, the Brazil nut oil coated with LT2 (the highest concentration of the tocopherol mix) showed a sharp drop in induction time, probably because, at 45 days of storage, the oil analyzed was more susceptible than the oil in the other samples to the heating process undergone during the test since, throughout the test and storage time, the oil undergoes various changes in the degree of unsaturation in its composition, so it can be inferred that during the 45-day storage period, there was a change in the degree of unsaturation of the oil which caused a shorter induction time, i.e. a decrease in the oxidative stability of the Brazil nuts coated with LT2.

Table 5 shows the results of the fit parameters of the zero-, first-, and second-order models of the oil of the Brazil nuts uncoated and coated of the coatings of cassava starch/CMC control B (no soy lecithin and no tocopherol mix), L (with soy lecithin and no tocopherol mix), and LT and LT2 (with soy lecithin and tocopherol mix) during storage time.

The zero-order and first-order models (Table 5) were the ones that best fit the experimental data for uncoated and B-coated Brazil nut oil, despite not having a good fit for the oil from Brazil nuts coated with L, LT, and LT2. However, Labuza [71] states that when the reaction is not sufficiently advanced (<50% conversion), both the zero-order model and the first-order model can be indistinguishable from the point of view of the fit. It is believed that the samples coated with L, LT, and LT2 had yet to reach the propagation stage of the oxidative process. Nevertheless, considering the R^2^ values for the two models reveals a trend for the LT and LT2 samples.

In addition, the models applied to the data from the accelerated oxidation test, in which Brazil nut oil without coating and with coatings B, L, LT, and LT2 were subjected to a temperature of 110 °C, make it possible to estimate the half-life of the oils. Checking the models that best fitted the Brazil nut oil data showed that the oil from uncoated nuts coated with B, LT, and LT2 had the most extended half-life. On the other hand, when considering the half-life of Brazil nut oil coated with LT and LT2 (added to the mixture of tocopherols), it was found that the LT coating showed the highest value of 130.74 h in the zero-order model.

The greater the amount of unsaturation, the more susceptible the oil is to degradation [72,73]. Thus, it can be said that the increase in temperature and exposure time during the test caused a decrease in the level of unsaturation present in the uncoated and coated Brazil nut oil, which is closely linked to the half-life since the degree of degradation of an oil can dictate the shelf life of a lipid product [73].

#### 3.4.5. Principal Component Analysis (PCA) and Hierarchical Cluster (HCA) of the Evaluation of the Oxidation Level of Oil of the Brazil Nuts Uncoated and Coated during Storage Time

**Figure 7:**
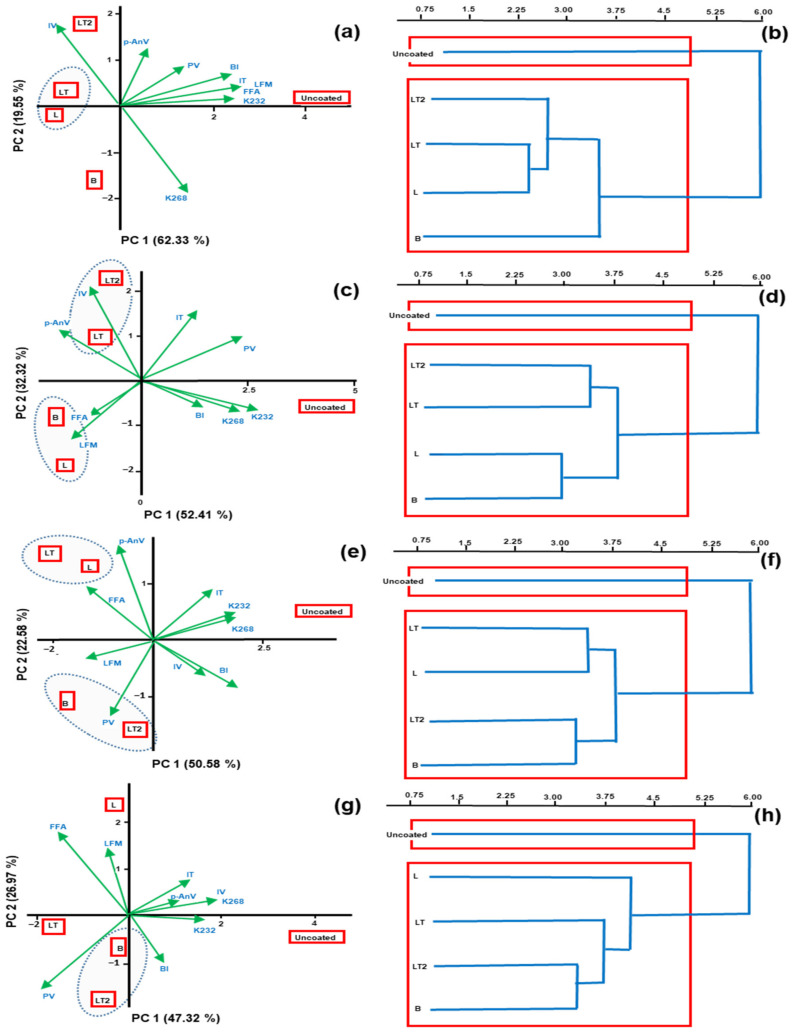

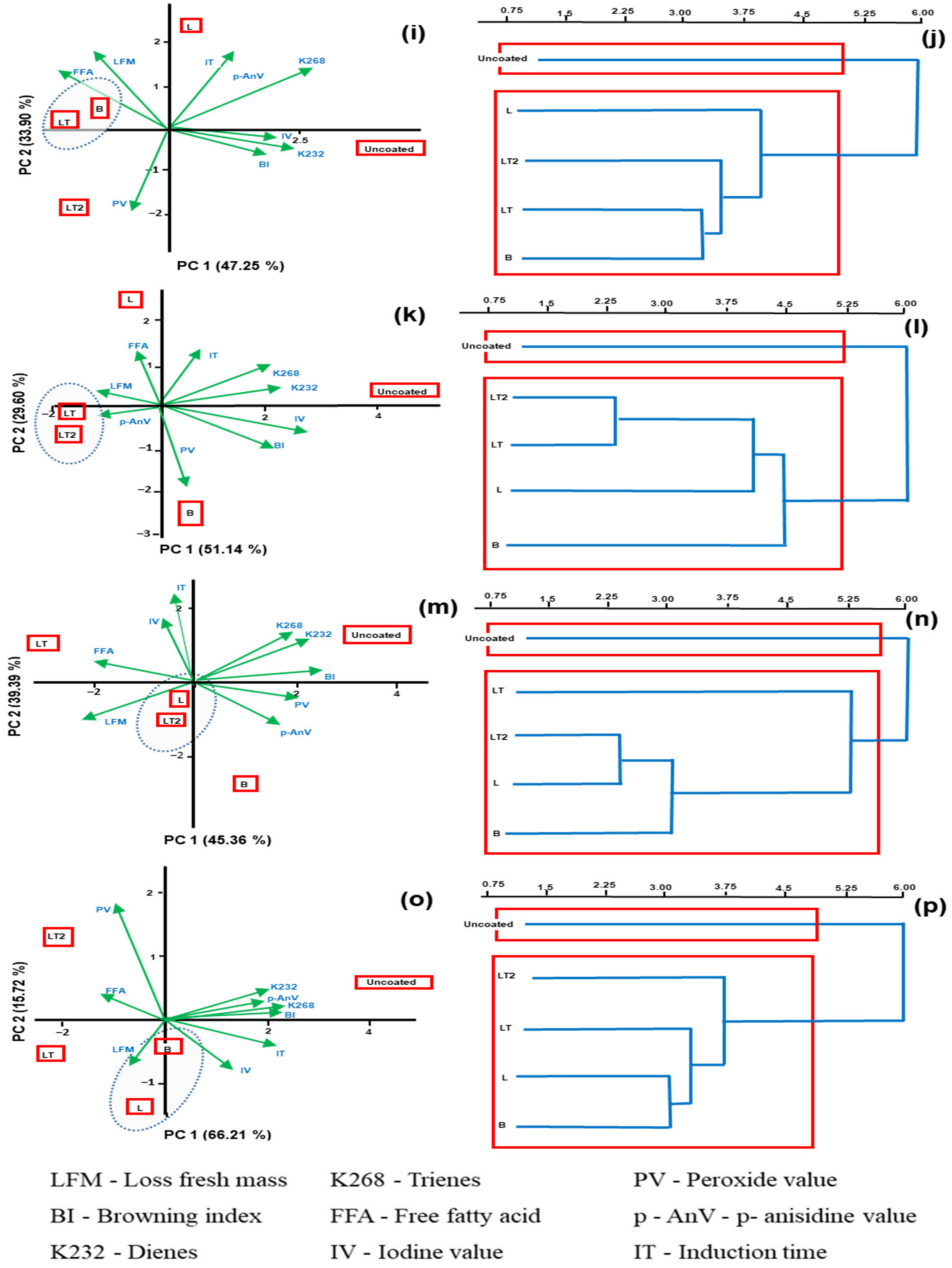
Biplot graphic of principal component analysis (**a**,**c**,**e**,**g**,**i**,**k**,**m**,**o**) on each storage day (1, 7, 15, 30, 45, 60, 90, and 120 days, respectively), and hierarchical cluster (**b**,**d**,**f**,**h**,**j**,**l**,**n**,**p**) on each storage day (1, 7, 15, 30, 45, 60, 90, and 120 days, respectively) of properties of the evaluation of the oxidation level of Brazil nuts uncoated and coated with coating of the cassava starch/CMC control B (no soy lecithin and no tocopherol mix), L (with soy lecithin and no tocopherol mix), and LT and LT2 (with soy lecithin and tocopherol mix) during storage time.

Figure 7 shows the biplot graphic of principal component analysis (PCA) and hierarchical cluster (HCA) of properties of the evaluation of the oxidation level of Brazil nuts uncoated and coated with coating of the cassava starch/CMC control B (no soy lecithin and no tocopherol mix), L (with soy lecithin and no tocopherol mix), and LT and LT2 (with soy lecithin and tocopherol mix) during storage time.

The variability explained by PCA (Figure 7a,c,e,g,i,k,m,o) was 81.88%, 84.73%, 73.16%, 74.29%, 81.15%, 80.74%, 84.75%, and 81.93% on each storage day (1, 7, 15, 30, 45, 60, 90, and 120 days, respectively). Furthermore, it can be observed that the oxidative indices contributed positively to the formation of the uncoated group on all days of storage, but at 7 and 60 days of storage evaluated, there was the formation of the LT-LT2 group, indicating similarity between the parameters. of Brazil nut oxidation during these days of storage of these samples. Corroborating the HCA (Figure 7b,d,f,h,j,l,n,p), which showed the formation of mainly uncoated clusters at all storage times and the formation of the LT-LT2 cluster observed at times 7 and 60 storage, demonstrating the similarity between the LT and LT2 parameters. Furthermore, observing the HCA, it can be seen that the clusters formed by the Euclidean distance present the formation of four clusters.

These results show that all the coatings applied to the Brazil nuts promoted the preservation of the samples in some of the evaluation periods. They also showed that most of the oxidative indices negatively influenced all the coated samples, especially the Brazil nuts coated with LT and LT2 coatings, which contained the tocopherol mix in their composition. That is an advantage, as it shows that the coatings generally protected the coated Brazil nuts, especially the Brazil nuts coated with LT and LT2.

## 4. Conclusions

In the rheological evaluation, the blend-forming solutions B, L, LT, and LT2 showed non-Newtonian and pseudoplastic behavior. All blend-forming solutions showed excellent resistance to flow and were stable. The pH of all the developed blend-forming solutions was close to neutral. The color of the blend-forming solutions tended slightly toward white, low (achromatic), revealing weak or diluted coloration and a slightly greenish-yellow tonality. Adding a tocopherol mix promoted a greater tonality in the blend-forming solutions LT and LT2.

The LT and LT2 coatings minimized the loss of fresh mass of Brazil nuts compared to B and L coatings but were higher when compared to uncoated Brazil nuts. The browning index of the coated Brazil nuts was lower than that of the uncoated Brazil nuts for almost the entire storage period; LT showed more stable values after 30 days of storage. Regarding the evaluation of the oxidative state of the uncoated and coated nuts, in general, the oil from the coated LT and LT2 Brazil nuts added to the tocopherol mix was preserved; the indices provided an insight into the possible minimization of oxidation of the Brazil nut oil under study. PCA showed that all coatings applied to Brazil nuts promoted oil preservation in some evaluation periods, especially the coatings added with a tocopherol mix. More research is needed. The first suggestion would be to determine the fatty acids in Brazil nut oil during the storage period to test the migration of the tocopherol mix into the headspace; the second suggestion is to apply it in the form of a film and the third is to apply it to other foods.

## Figures and Tables

**Table 1:** Zero-order, first-order, and second-order kinetic models.

Reaction	Model	Equation Number
Zero	$A=A_{0}-k\theta$	(9)
First	$lnA={lnA}_{0}-k\theta$	(10)
Second	$1/A=k\theta +1/A_{0}$	(11)

A—concentration of the evaluated parameter after a time θ; A_0_—initial concentration of the evaluated parameter; k—reaction speed constant; θ—time.

**Table 2:** Mean ± standard deviation of the fit parameters of the Newton, power law, and Herschel–Bulkley models to the rheological behavior of the blend-forming solutions B (no soy lecithin and no tocopherol mix), L (with soy lecithin and no tocopherol mix), and LT and LT2 (with soy lecithin and tocopherol mix).

Blend-Forming Solutions of the Coatings	Fitting Parameters—Newton’s Model					
	n	R^2^		χ^2^		RMSE
B	0.028 ± 0.0001 c	0.998 ± 0.0001 a		0.004 ± 0.0001 c		0.310 ± 0.004 c
L	0.116 ± 0.0004 a	0.979 ± 0.0004 c		0.811 ± 0.0100 a		4.461 ± 0.10 a
LT	0.011 ± 0.0001 d	0.992 ± 0.0004 b		0.003 ± 0.0001 c		0.273 ± 0.01 c
LT2	0.074 ± 0.0001 b	0.993 ± 0.0001 b		0.117 ± 0.001 b		1.674 ± 0.005 b
*p*-Value homoscedasticity	<0.001	0.005		0.038		0.008
*p*-Value ANOVA/Welch	<0.001	<0.001		<0.001		<0.001
Blend-forming solutions of the coatings	**Fitting Parameters—Power Law model**					
	**K (mPa·s** **^n^** **)**	**n**		**R** **^2^**	**χ** **^2^**	**RMSE**
B	0.151 ± 0.190 ab	0.945 ± 0.001 a		0.999 ± 0.00001 a	0.012 ± 0.00001 c	0.052 ± 0.00006 c
L	0.282 ± 0.002 a	0.816 ± 0.009 c		0.999 ± 0.00001 a	0.024 ± 0.00001 b	0.766 ± 0.00200 a
LT	0.021 ± 0.0001 b	0.883 ± 0.0001 b		0.999 ± 0.00001 a	0.134 ± 0.00001 a	0.017 ± 0.00002 d
LT2	0.128 ± 0.0001 ab	0.890 ± 0.001 b		0.999 ± 0.00001 a	0.002 ± 0.00001 d	0.247 ± 0.00800 b
*p*-Value homoscedasticity	<0.001	0.001		0.001	0.005	0.018
*p*-Value ANOVA/Welch	<0.001	<0.001		<0.001	<0.001	<0.001
Blend-forming solutions of the coatings	**Fitting Parameters—Herschel–Bulkley model**					
	**σ (Pa)**	**K (mPa.s** **^n^** **)**	**n**	**R** **^2^**	**χ** **^2^**	**RMSE**
B	0.000 ± 0.008 a	0.037 ± 0.00001 c	0.945 ± 0.00001 a	0.999 ± 0.00001 a	1.200 ± 0.002 c	0.002 ± 0.001 d
L	0.000 ± 0.140 a	0.285 ± 0.00300 a	0.816 ± 0.00001 c	0.999 ± 0.00001 a	0.027 ± 0.004 a	0.580 ±0.004 b
LT	0.000 ± 0.003 a	0.020 ± 3.43000 d	0.878 ± 0.00001 b	0.999 ± 0.00100 a	1.780 ± 0.001 b	3.070 ± 0.016 a
LT2	0.001 ± 0.004 a	0.125 ± 0.00001 b	0.890 ± 0.00001 b	0.999 ± 0.00001 a	0.003 ± 0.0001 d	0.078 ± 0.001 c
*p*-Value homoscedasticity	<0.001	<0.001	0.003	<0.001	0.026	0.012
*p*-Value ANOVA/Welch	<0.001	<0.001	<0.001	<0.001	<0.001	<0.001

Means followed by the same lowercase letter in each column did not differ significantly by Tukey’s test at the 5% level. K = consistency index, n = fluid behavior index, R^2^ = correlation coefficient, χ^2^ = chi-squared, RMSE = root mean squared error, σ (Pa) = residual stress.

**Table 3:** Mean ± standard deviation of the pH, color parameters, and antioxidant activity of the blend-forming solutions B (no soy lecithin and no tocopherol mix), L (with soy lecithin and no tocopherol mix), and LT and LT2 (with soy lecithin and tocopherol mix).

Blend-Forming Solutions of the Coatings	pH	Luminosity (L*)	Chromaticity (a*)	Chromaticity (b*)	Chroma (C*)	Hue Angle (h°)	Antioxidant Activity (by DPPH• Method) (%)
B	6.05 ± 0.03 c	12.93 ± 0.58 c	−1.34 ± 0.01 a	7.36 ± 0.01 ab	7.29 ± 0.04 ab	100.04 ± 0.08 c	1.69 ± 0.01 c
L	6.22 ± 0.005 b	13.08 ± 0.03 c	−1.35 ± 0.01 a	7.65 ± 0.16 a	7.53 ± 0.31 a	99.32 ± 0.41 d	8.66 ± 0.01 d
LT	6.22 ± 0.005 b	15.08 ± 0.02 b	−1.44 ± 0.01 b	6.72 ± 0.03 bc	6.88 ± 0.03 bc	102.37 ± 0.01 b	58.40 ± 0.01 b
LT2	6.53 ± 0.008 a	16.61 ± 0.07 a	−1.63 ± 0.02 c	6.46 ± 0.04 c	6.66 ± 0.03 c	103.90 ± 0.02 a	81.69 ± 0.05 a
*p*-Value homoscedasticity	0.045	0.002	0.424	0.009	0.008	0.005	0.056
*p*-Value ANOVA/ Welch	<0.001	<0.001	<0.001	<0.001	<0.001	<0.001	<0.001

Means followed by the same lowercase letter in each column did not differ significantly by Tukey’s test at the 5% level.

**Table 4:** Mean ± standard deviation of the quality parameters and induction time of the oil of the Brazil nuts uncoated and coated with the coatings of cassava starch/CMC control B (no soy lecithin and no tocopherol mix), L (with soy lecithin and no tocopherol mix), and LT and LT2 (with soy lecithin and tocopherol mix) during storage time.

Parameters	Brazil Nuts Coated and Uncoated	Storage Time (Days)							
		1	7	15	30	45	60	90	120
K232	Uncoated	0.12 ± 0.005 aE	2.16 ± 0.03 aD	2.45 ± 0.03 aC	2.67 ± 0.003 aB	2.67 ± 0.02 aB	2.68 ± 0.02 aB	2.69 ± 0.0001 aB	4.46 ± 0.0001 aA
	B	0.12 ± 0.005 aB	0.12 ± 0.006 bB	0.11 ± 0.01 bB	0.12 ± 0.0001 bE	0.14 ± 0.0001 cA	0.12 ± 0.005 cB	0.12 ± 0.0001 eB	0.11 ± 0.005 dB
	L	0.12 ± 0.0001 aF	0.12 ± 0.005 bEF	0.10 ± 0.0001 bG	0.13 ± 0.005 bE	0.23 ± 0.0001 bB	0.18 ± 0.0001 bC	0.25 ± 0.005 bA	0.15 ± 0.0001 bD
	LT	0.12 ± 0.0001 aB	0.12 ± 0.0001 bB	0.10 ± 0.005 bC	0.12 ± 0.01 bB	0.12 ± 0.005 cB	0.12 ± 0.006 cB	0.18 ± 0.0001 cA	0.12 ± 0.0001 cdB
	LT2	0.12 ± 0.0001 aB	0.12 ± 0.0001 bB	0.10 ± 0.0001 bD	0.13 ± 0.0001 bA	0.13 ± 0.005 cA	0.13 ± 0.0001 cA	0.13 ± 0.005 dA	0.11 ± 0.006 dC
*p*-Value homoscedasticity		<0.001	0.024	0.004	0.001	0.004	0.004	<0.001	<0.001
*p*-Value ANOVA/Welch		<0.001	<0.001	<0.001	<0.001	<0.001	<0.001	<0.001	<0.001
K268	Uncoated	0.12 ± 0.0001 aG	0.29 ± 0.0001 aD	0.24 ± 0.0001 aF	0.25 ± 0.005 aE	0.28 ± 0.01 aD	0.35 ± 0.0001 aC	0.37 ± 0.002 aB	0.63 ± 0.0001 aA
	B	0.12 ± 0.005 aA	0.12 ± 0.0001 bA	0.11 ± 0.0001 bcB	0.12 ± 0.0001 bA	0.12 ± 0.0001 cA	0.11 ± 0.0002 eB	0.12 ± 0.0001 dA	0.12 ± 0.005 dA
	L	0.12 ± 0.006 aD	0.12 ± 0.005 bD	0.12 ± 0.005 bD	0.13 ± 0.005 bC	0.23 ± 0.005 bB	0.23 ± 0.0001 bB	0.25 ± 0.005 bA	0.25 ± 0.0001 bA
	LT	0.12 ± 0.0001 aA	0.12 ± 0.0001 bA	0.12 ± 0.001 bA	0.12 ± 0.005 bA	0.12 ± 0.003 cA	0.12 ± 0.0001 dA	0.12 ± 0.0001 dA	0.12 ± 0.0001 dA
	LT2	0.12 ± 0.005 aB	0.11 ± 0.005 bcC	0.12 ± 0.001 bB	0.12 ± 0.005 bB	0.12 ± 0.0001 cB	0.13 ± 0.0001 cA	0.13 ± 0.0001 cA	0.13 ± 0.006 cA
*p*-Value homoscedasticity		0.004	<0.001	<0.001	0.034	0.056	<0.001	<0.001	<0.001
*p*-Value ANOVA/Welch		<0.001	<0.001	<0.001	<0.001	<0.001	<0.001	<0.001	<0.001
FFA (mg KOH·g^−1^)	Uncoated	1.63 ± 0.00001 aA	1.63 ± 0.00001 cA	1.46 ± 0.02 dDE	1.51 ± 0.02 bC	1.56 ± 0.01 bB	1.50 ± 0.03 aCD	1.32 ± 0.01 dF	1.44 ± 0.01 cE
	B	1.63 ± 0.001 aB	3.38 ± 0.01 aA	3.17 ± 0.02 bA	3.55 ± 0.8 aA	3.71 ± 0.8 aA	1.51 ± 0.06 aB	1.48 ± 0.01 cdB	1.55 ± 0.01 cB
	L	1.63 ± 0.001 aB	3.25 ± 0.01 bA	3.28 ± 0.02 aA	3.38 ± 0.14 aA	3.27 ± 0.19 aA	1.93 ± 0.8 aB	1.42 ± 0.02 cB	1.57 ± 0.02 cB
	LT	1.63 ± 0.001 aE	3.26 ± 0.08 bBC	3.31 ± 0.02 aBC	3.32 ± 0.07 aB	3.14 ± 0.04 aC	1.55 ± 0.03 aE	2.89 ± 0.01 aD	4.20 ± 0.0001 aA
	LT2	1.63 ± 0.008 aB	1.64 ± 0.03 cB	1.52 ± 0.01 cB	1.56 ± 0.14 bB	1.59 ± 0.03 bB	1.51 ± 0.05 aB	1.61 ± 0.09 bCB	3.07 ± 0.14 bA
*p*-Value homoscedasticity		0.001	0.016	0.857	<0.001	<0.001	<0.001	0.003	0.042
*p*-Value ANOVA/Welch		0.324	<0.001	<0.001	<0.001	<0.001	0.599	<0.001	<0.001
IV (gI_2_·100 g^−1^)	Uncoated	59.1 ± 0.03 aE	54.6 ± 0.03 dG	82.8 ± 0.01 aB	102.6 ± 0.08 aA	81.8 ± 0.02 aB	74.5 ± 0.03 aD	78.2 ± 0.04 aC	56.6 ± 0.04 aF
	B	59.2 ± 0.05 aDE	58.8 ± 0.03 cDE	84.4 ± 0.20 aB	88.4 ± 0.03 bA	60.8 ± 0.06 bD	69.8 ± 0.02 bC	58.1 ± 0.06 cE	52.9 ± 0.06 bF
	L	59.2 ± 0.10 aDE	79.1 ± 0.15 aA	60.0 ± 0.05 bCDE	63.4 ± 0.02 cC	57.5 ± 0.02 cE	62.6 ± 0.24 cCD	66.4 ± 0.23 bB	41.0 ± 0.02 dF
	LT	59.3 ± 0.09 aBC	52.1 ± 0.15 eCD	47.8 ± 0.60 cDE	51.0 ± 0.09 dDE	48.1 ± 0.005 dDE	60.0 ± 0.30 cB	82.4 ± 0.08 aA	44.7 ± 0.27 cE
	LT2	59.5 ± 0.11 aAB	60.9 ± 0.14 bAB	53.9 ± 0.05 bcB	54.9 ± 0.35 dAB	57.2 ± 0.16 cAB	62.2 ± 0.03 cA	57.7 ± 0.60 cAB	25.4 ± 0.04 eC
*p*-Value homoscedasticity		0.188	0.002	0.001	0.007	0.007	0.004	0.059	0.002
*p*-Value ANOVA/Welch		0.079	<0.001	<0.001	<0.001	<0.001	<0.001	<0.001	<0.001
PV (meq·kg^−1^)	Uncoated	2.33 ± 0.01 aG	3.04 ± 0.03 abF	3.07 ± 0.005 abEF	3.09 ± 0.01 dE	4.54 ± 0.01 bcD	5.78 ± 0.01 cC	11.21 ± 0.005 bB	11.93 ± 0.02 cA
	B	2.30 ± 0.01 aF	2.55 ± 0.04 abF	3.26 ± 0.24 abE	3.49 ± 0.01 bE	4.82 ± 0.5 bD	11.32 ± 0.005 aC	12.09 ± 0.005 aB	13.12 ± 0.04 bA
	L	2.28 ± 0.03 aE	2.54 ± 0.42 bE	3.01 ± 0.01 bD	3.21 ± 0.005 cD	3.81 ± 0.01 dC	4.67 ± 0.01 dB	10.61 ± 0.07 cA	10.95 ± 0.04 eA
	LT	2.32 ± 0.03 aH	3.02 ± 0.01 abG	3.21 ± 0.07 abF	3.54 ± 0.06 bE	4.21 ± 0.14 cdD	4.69 ± 0.01 dC	7.28 ± 0.02 eB	11.59 ± 0.03 dA
	LT2	2.32 ± 0.03 aH	3.06 ± 0.01 aG	3.33 ± 0.005 aF	3.83 ± 0.01 aE	5.55 ± 0.12 aD	6.10 ± 0.01 bC	9.64 ± 0.01 dB	17.70 ± 0.02 aA
*p*-Value homoscedasticity		0.107	<0.001	0.024	0.013	0.002	0.584	0.008	0.641
*p*-Value ANOVA/Welch		0.265	<0.001	<0.001	<0.001	<0.001	<0.001	<0.001	<0.001
p-AnV	Uncoated	7.77 ± 0.01 aA	4.37 ± 0.03 dG	6.28 ± 0.02 bD	6.29 ± 0.01 bC	5.92 ± 0.01 bE	4.30 ± 0.005 dH	5.05 ± 0.006 cF	7.11 ± 0.07 aB
	B	7.66 ± 1.16 aA	5.38 ± 0.25 bE	6.27 ± 0.02 bB	6.28 ± 0.02 bcB	5.61 ± 0.08 cD	4.48 ± 0.52 cF	5.63 ± 0.03 aD	5.87 ± 0.06 bC
	L	7.77 ± 0.01 aA	5.12 ± 0.74 cE	6.23 ± 0.02 cB	6.25 ± 0.02 cB	6.01 ± 0.01 aC	4.80 ± 0.02 bF	5.05 ± 0.23 cE	5.58 ± 0.64 cD
	LT	7.74 ± 0.01 aA	5.07 ± 0.04 cC	6.73 ± 0.02 aB	6.74 ± 0.01 aB	3.56 ± 0.02 eG	4.85 ± 0.09 bD	4.49 ± 0.02 dF	4.55 ± 0.02 eE
	LT2	7.77 ± 0.57 aA	5.74 ± 0.03 aC	6.11 ± 0.01 dB	6.09 ± 0.28 dB	4.11 ± 0.01 dF	5.41 ± 0.27 aD	5.17 ± 0.01 bE	5.47 ± 0.17 dD
*p*-Value homoscedasticity		<0.001	<0.001	0.010	<0.001	0.064	0.002	<0.001	0.002
*p*-Value ANOVA/Welch		<0.001	<0.001	<0.001	<0.001	<0.001	<0.001	<0.001	<0.001
TOTOX	Uncoated	12.43 ± 0.02 aE	10.46 ± 0.06 bF	12.41 ± 0.02 bE	12.47 ± 0.03 dE	15.00 ± 0.02 aD	15.86 ± 0.01 cC	27.47 ± 0.01 bB	30.96 ± 0.10 cA
	B	12.27 ± 1.14 aE	10.48 ± 0.31 bF	12.78 ± 0.49 abE	13.26 ± 0.03 bE	15.26 ± 0.92 aD	27.12 ± 0.51 aC	29.81 ± 0.04 aB	32.12 ± 0.07 bA
	L	12.33 ± 0.06 aD	10.20 ± 0.77 bE	12.25 ± 0.03 bD	12.66 ± 0.03 cD	13.63 ± 0.02 bC	14.14 ± 0.04 eC	26.27 ± 0.17 cB	27.49 ± 0.58 eA
	LT	12.38 ± 0.06 aF	11.12 ± 0.06 abH	13.15 ± 0.14 aE	13.83 ± 0.10 aD	11.98 ± 0.31 cG	14.23 ± 0.07 dC	19.05 ± 0.07 eB	27.73 ± 0.08 dA
	LT2	12.40 ± 0.49 aG	11.85 ± 0.01 aH	12.78 ± 0.09 abF	13.76 ± 0.26 aE	15.22 ± 0.23 aD	17.62 ± 0.26 bC	24.45 ± 0.01 dB	40.87 ± 0.16 aA
*p*-Value homoscedasticity		<0.001	0.002	0.029	0.003	0.002	0.002	0.061	0.002
*p*-Value ANOVA/Welch		0.023	<0.001	<0.001	<0.001	<0.001	<0.001	<0.001	<0.001

Means followed by the same uppercase letter in each row and lowercase letter in each column do not differ significantly by Tukey’s test at the 5% level. K232—dienes, K268—trienes, FFA—free fatty acid, IV—iodine value, PV—peroxide value, p-AnV—p-anisidine value, TOTOX—total oxidation.

**Table 5:** Mean ± standard deviation of the fit parameters of the zero-, first-, and second-order models of oil of the Brazil nuts uncoated and coated of the coatings of cassava starch/CMC control B (no soy lecithin and no tocopherol mix), L (with soy lecithin and no tocopherol mix), and LT and LT2 (with soy lecithin and tocopherol mix) during storage time.

Oil of Brazil Nuts Coated and Uncoated	Fitting Parameters—Zero-Order Model					
	A0	k	R^2^	χ^2^	RMSE	θ (1/2)
Uncoated	12.58	0.044	0.838	0.220	0.801	142.02
B	10.17	0.043	0.909	0.060	0.393	117.49
L	11.25	0.031	0.311	0.528	4.739	176.99
LT	10.22	0.039	0.585	0.073	2.290	130.74
LT2	10.64	0.044	0.641	0.001	2.354	119.28
	**Fitting Parameters—First-Order Model**					
	A0	k	R^2^	χ^2^	RMSE	θ (1/2)
Uncoated	12.96	0.005	0.789	0.0257	0.013	141.42
B	10.65	0.006	0.834	0.0157	0.017	108.28
L	11.54	0.004	0.368	0.0578	0.066	161.16
LT	10.74	0.006	0.584	0.0005	0.056	113.60
LT2	11.39	0.007	0.607	0.0135	0.070	96.25
	**Fitting Parameters—Second-Order Model**					
	A0	k	R^2^	χ^2^	RMSE	θ (1/2)
Uncoated	13.69	0.006	0.735	0.068	0.291	12.16
B	11.76	0.001	0.752	0.018	0.038	85.00
L	12.19	0.006	0.416	0.065	0.280	13.66
LT	12.09	0.001	0.570	0.020	0.036	82.70
LT2	13.77	0.001	0.571	0.075	0.290	55.84

A0 = initial concentration of the evaluated parameter; k = rate constants; R^2^ = correlation coefficient; χ^2^ = chi-squared; RMSE = root mean square error; θ (1/2) = half-life time.

## Data Availability

The original contributions presented in the study are included in the article, further inquiries can be directed to the corresponding author.
